# Gene expression changes leading extreme alkaline tolerance in Amur ide (*Leuciscus waleckii)* inhabiting soda lake

**DOI:** 10.1186/1471-2164-14-682

**Published:** 2013-10-04

**Authors:** Jian Xu, Qiang Li, Liming Xu, Shaolin Wang, Yanliang Jiang, Zixia Zhao, Yan Zhang, Jiongtang Li, Chuanju Dong, Peng Xu, Xiaowen Sun

**Affiliations:** 1 Centre for Applied Aquatic Genomics, Chinese Academy of Fishery Sciences, Beijing 100141, China; 2 Department of Psychiatry & Neurobiology Science, University of Virginia, Charlottesville, VA 22911, USA; 3 College of Life Science and Technology, Shanghai Ocean University, Shanghai 201306, China

**Keywords:** *L. waleckii*, RNA-Seq, Gene expression, Adaptation

## Abstract

**Background:**

Amur ide (*Leuciscus waleckii*) is an economically and ecologically important cyprinid species in Northern Asia. The Dali Nor population living in the soda lake Dali Nor can adapt the extremely high alkalinity, providing us a valuable material to understand the adaptation mechanism against extreme environmental stress in teleost.

**Results:**

In this study, we generated high-throughput RNA-Seq data from three tissues gill, liver and kidney of *L. waleckii* living in the soda lake Dali Nor and the fresh water lake Ganggeng Nor, then performed parallel comparisons of three tissues. Our results showed that out of assembled 64,603 transcript contigs, 28,391 contigs had been assigned with a known function, corresponding to 20,371 unique protein accessions. We found 477, 2,761 and 3,376 differentially expressed genes (DEGs) in the gill, kidney, and liver, respectively, of Dali Nor population compared to Ganggeng Nor population with FDR ≤ 0.01and fold-change ≥ 2. Further analysis revealed that well-known functional categories of genes and signaling pathway, which are associated with stress response and extreme environment adaptation, have been significantly enriched, including the functional categories of “response to stimulus”, “transferase activity”, “transporter activity” and “oxidoreductase activity”, and signaling pathways of “mTOR signaling”, “EIF2 signaling”, “superpathway of cholesterol biosynthesis”. We also identified significantly DEGs encoding important modulators on stress adaptation and tolerance, including carbonic anhydrases, heat shock proteins, superoxide dismutase, glutathione S-transferases, aminopeptidase N, and aminotransferases.

**Conclusions:**

Overall, this study demonstrated that transcriptome changes in *L. waleckii* played a role in adaptation to complicated environmental stress in the highly alkalized Dali Nor lake. The results set a foundation for further analyses on alkaline-responsive candidate genes, which help us understand teleost adaptation under extreme environmental stress and ultimately benefit future breeding for alkaline-tolerant fish strains.

## Background

Amur ide (*Leuciscus waleckii*) belongs to the family of cyprinid, inhabiting the Heilongjiang (Amur) River basin in Russia, Mongolia, China and Korea. Although *L. waleckii* inhabits fresh water in rivers, streams and lakes, it also has great tolerance on high salinity and alkalinity (http://www.fishbase.org). As an extreme instance, *L. waleckii* inhabiting Dali Nor lake, Inner Mongolia (E116^o^25′-116^o^45′,N43^o^13′-43^o^23′) can survive in water of ultra-high alkalinity up to pH 9.6. Dali Nor lake is a typical saline-alkaline lake with high concentrations of carbonate salts. It locates in an endorheic basin on eastern Inner Mongolia Plateau. The evaporation is greater than precipitation and inflows, making the lake shrink consistently from 1600 to less 200 km^2^ since early Holocene (11,500–7,600 cal yr BP). The alkalinity and salinity are increasing steadily [1]. Currently the pH value ranged from 8.25 to 9.6, with the alkaline content (ALK) over 50 mg/L and the salinity around 6 ‰. Combining geological and biological evidence, it’s commonly believed that *L. waleckii* population in Dali Nor lake were used to be fresh water fish that evolved fast in the past several thousand years and developed great tolerance on high alkalinity [2,3].

*L. waleckii* is economically important to local Mongolian who live around the Dali Nor lake, and ecologically important to wild birds on their migration journeys from Siberia to the south which feed on *L. waleckii* as major food source [4]. In spite of economic and ecological importance, the mechanism of its high tolerance on alkalinity is still a puzzle. Very limited physiological and genetic studies had been performed, and rare genetic resources had been developed. So far, only a few genetic markers had been developed for population genetics evaluation and phylogeny analysis [3,5]. Mitochondrial genome had been completely sequenced and annotated, providing basic molecular tools for ecological and genetic study [6]. Scientists are paying more attention to *L. waleckii* with gradually recognized importance. Recently, high throughput transcriptome sequencing was performed on Illumina platform and analyzed, providing the genomic basis for further investigation of the mechanism of its alkaline tolerance [7]. *L. waleckii* has been recently developed as potential aquaculture species in the widely distributed saline and alkaline water in northern China. The breeding program also eagerly desires better understanding of its physiological and genetic basis of the tolerance adaptation and stress resistant on alkaline environment. Besides, scientists are also interested on the mechanism of microevolution on *L. waleckii* which evolved fast to adapt paleoenvironmental changes since early Holocene.

Comparative study between organisms inhabiting distinct environments could provide insight into the mechanism that responding to the environmental difference. In some cases, scientists apply artificial treatments to create the difference in the experiments, and facilitate the comparison [8,9]. To better understand the physiological and genetic changes and mechanism of alkaline tolerance and adaptation in *L. waleckii,* comparative analysis between the fish living in alkaline water and fresh water is the efficient method. Fortunately, there is a sister lake of Dali Nor called Ganggeng Nor, which is fresh water lake and connected to Dali Nor through the short Shali river. *L. waleckii* also inhabits the fresh water of Ganggeng Nor lake. There is frequent genetic communication between the population in Dali Nor lake (alkaline water type, AW) and those in Ganggeng Nor lake (fresh water type, FW) through anadromous spawning migration annually. Both types of *L. waleckii* are derived from same ancestors and have consistent genetic background, which provide us unique natural samples to explore gene expression changes in response to high alkaline environment.

Transcriptome profiling and differential gene expression analyses traditionally use microarray technology, which requires cDNA library, Expressed Sequence Tags (EST) dataset and array hybridization. With the emerging of the next generation sequencing, RNA sequencing (RNA-Seq) is relatively new technology for transcriptomic study across the whole genome. Comparing to traditional cDNA microarray, RNA-Seq provides deep sequencing data for direct quantification of transcripts, which is more sensitive to detect all expressed genes without the hassles of EST collection, probe synthesis, microarray design and hybridization [10,11]. In the past several years, RNA-Seq has been widely used in many teleost for differential gene expression analysis in various organisms. For instance, RNA-Seq were used to unveil gene expression differences in response to various pathogenic challenge in *Lateolabrax japonicas*[12], catfish (*Ictalurus punctatus*) [13,14], Grouper (*Epinephelus spp.*) [15], European sea bass (*Dicentrarchus labrax*) [16] and Asian sea bass (*Lates calcarifer*) [8]. It was even used to quantify the gene expression changes in *Fundulus grandis* in the Gulf of Mexico to evaluate the impact of oil contamination after the disaster of Deepwater Horizon drilling platform [17]. Gene expression changes responding to abiotic stress are generally very significant comparing to those control counterparts. Thus, RNA-Seq was also used to profile DEGs and pathways under certain environmental stress. For instance, drought-responsive genes were identified and analyzed using RNA-Seq to compare drought-treated and well-watered fertilized ovary and basal leaf meristem tissue [18]. Gene expression changes in response to extreme dehydration on *Belgica Antarctica* were characterized using RNA-Seq, unveiling the tolerance mechanisms on dehydration in Antarctic insect [19]. RNA-Seq results also revealed gene expression changes in various metabolic pathways in response to osmotic stress and exogenous abscisic acid challenge, providing global gene expression overview of drought stress sorghum [20].

In this study, we use RNA-Seq to investigate the genome-wide gene expression differences in *L. waleckii* population inhabiting soda water of Dali Nor lake and their sister population inhabiting fresh water of Ganggeng Nor lake. Gene expression changes are identified from whole transcriptome background. Our study highlights those reactive pathways in response to high alkaline stress by using gene ontology and pathway analysis. This study provides us useful information to explain mechanism of alkaline stress tolerance in teleost.

## Results and discussion

### RNA-Seq data processing, reference assembly and alignment

To provide comprehensive understanding of the expression difference between *L. waleckii* inhabiting AW and FW, we collected and deeply sequenced the RNA samples from liver, kidney and gill. A total of 187,430,252 paired-end reads were generated from six samples with 101-bp read length. The number of sequences from each sample ranged from 28.7 to 35.7 million. After removal of ambiguous nucleotides, low-quality sequences (Phred quality scores < 20), contaminated microbial sequences, ribosomal RNA sequences, a total of 154,265,700 cleaned reads (82.3%) were harvested for further analysis. The cleaned sequences of each sample ranged from 23.7 to 29.1 million reads, showing the stability and consistence on sampling, library preparation and sequencing. Cleaned RNA-Seq reads of six samples were mapped to assembled transcriptome reference by using the ultrafast short read aligner Bowtie (version 0.12.3) [21]. The mapping ratio ranged from 80.8% to 88.1% with an average of 84.1%. All RNA-Seq data in this study have been deposited in the NCBI SRA database (Accession number SRR949612) (Table 1).

**Table 1:** Summary of samples and RNA-Seq data

**Group**	**Tissue**	**Reads**	**Clean reads**	**Mapped reads**	**Mapping ratio (%)**
FW	Liver	35,709,347	29,102,462	25,626,600	88.1
	Kidney	32,477,574	25,739,081	20,784,208	80.8
	gill	30,928,282	24,721,200	21,141,048	85.5
AW	Liver	28,660,883	23,733,500	19,620,262	82.7
	Kidney	29,645,565	24,974,142	21,061,309	84.3
	gill	30,008,601	25,995,315	21,628,548	83.2
Total		187,430,252	154,265,700		

**Figure 1:**
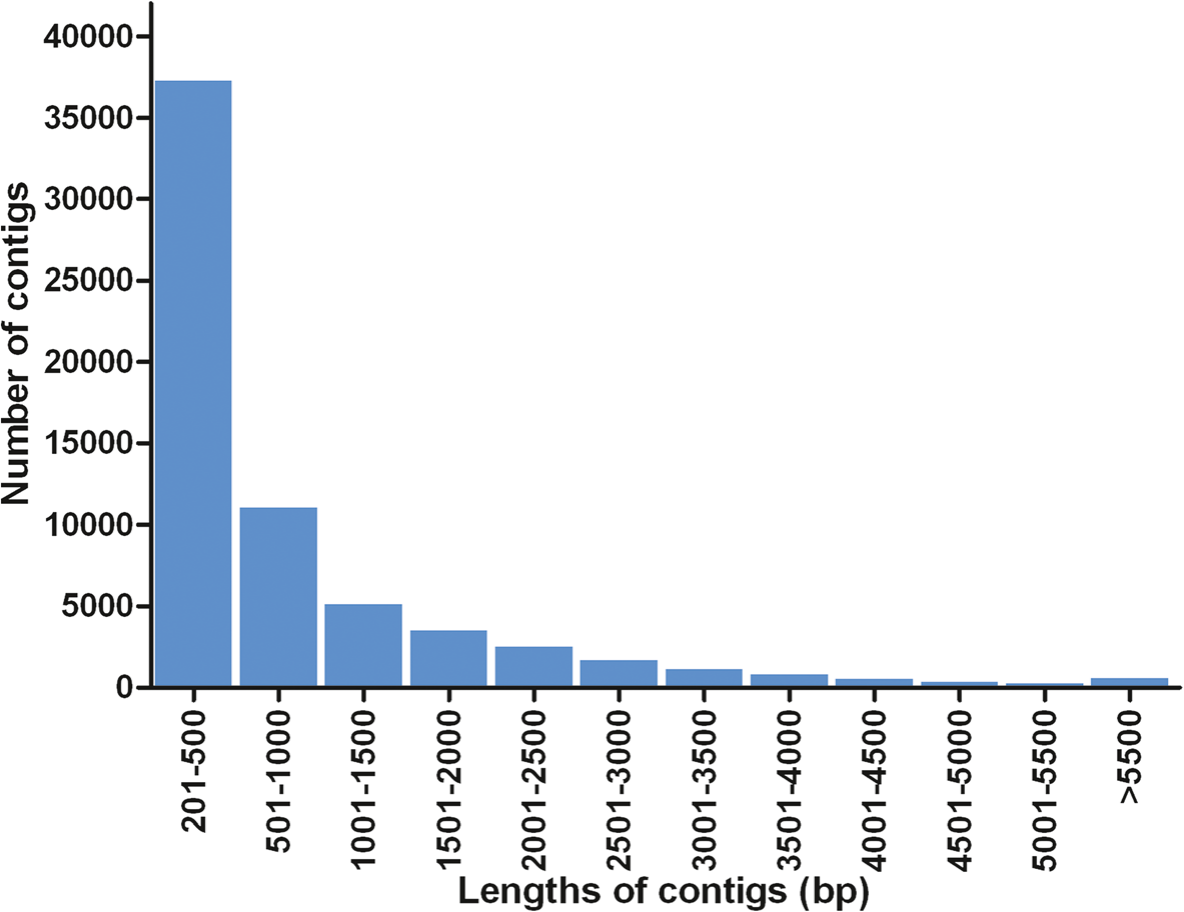
**Length distribution of assembled transcriptome contigs of *L. waleckii*.**

The cleaned reads of six samples were pooled and assembled by using Trinity assembler [22] to generate the transcriptome reference. As shown in Table 2, the trancriptome were assembled into 64,603 contigs, ranging from 201 to 16,177 bp in length. The average length is 879 bp, N50 length is 1,776 bp and median length is 404 bp. The contig length distribution was shown in Figure 1. We then annotated assembled contigs to provide expression background and facilitate the functional analysis of DEGs. We compared our assembly with three protein databases, including NCBI non-redundant (nr) protein database, uniprot database, and zebrafish reference protein database, by using BLASTx with e-value cutoff of 1e^-10^. A total of 28,391 contigs have significant hit at least in one database, corresponding to 20,371 unique protein accessions (Table 2). Gene ontology (GO) analysis was conducted to assign GO term to each of those 20,371 unique proteins. A total of 14,326 unique proteins were assigned at least one GO term for describing biological processes, molecular functions and cellular components, corresponding to 22,460 assembled contigs (Table 3).

**Table 2:** Statistics of transcriptome reference assembly and annotation

		
**Assembly**	Number of contigs	64,603
	Maximum contig length	16,177 bp
	Minimum contig length	201 bp
	Average contig length	879 bp
	Median contig length	404 bp
	N50 length	1,776 bp
**Annotation**	Contigs with blast hits on NR	27,724
	Contigs with blast hits on Uniprot	23,111
	Contigs with blast hits on *D.rerio* protein	26,611
	Unigenes with blast results	20,371
	Contigs with GO terms	22,460
	Unigenes with GO terms	14,326

**Table 3:** GO enrichment analysis of genes up- or down-regulated in response to AW stress

**Tissue**	**Go term**	**Definition**	**No. up- or down- regulated**	**Total in category**	**P value**
Liver	Up				
	**GO:0016491**	oxidoreductase activity	52	616	0.001
	**GO:0005215**	transporter activity	139	690	0.001
	**GO:0008047**	enzyme activator activity	30	141	0.019
	**GO:0045182**	translation regulator activity	1	65	0.009
	**GO:0002376**	immune system process	70	219	0.001
	**GO:0008152**	metabolic process	686	5,566	0.010
	Down				
	**GO:0016740**	transferase activity	201	1,555	0.002
	**GO:0009055**	electron carrier activity	28	99	0.001
	**GO:0016491**	oxidoreductase activity	132	616	0.000
	**GO:0030234**	enzyme regulator activity	77	506	0.001
	**GO:0009056**	catabolic process	139	1,061	0.007
	**GO:0009058**	biosynthetic process	216	1,791	0.027
Kidney	Up				
	**GO:0016491**	oxidoreductase activity	150	616	0.000
	**GO:0016740**	transferase activity	165	1,555	0.000
	**GO:0016829**	lyase activity	32	121	0.000
	**GO:0005215**	transporter activity	77	690	0.005
	**GO:0009055**	electron carrier activity	32	99	0.000
	**GO:0030234**	enzyme regulator activity	56	506	0.001
	**GO:0050896**	response to stimulus	91	678	0.000
	Down				
	**GO:0016491**	oxidoreductase activity	41	616	0.000
	**GO:0005215**	transporter activity	124	690	0.000
	**GO:0030234**	enzyme regulator activity	93	506	0.001
	**GO:0003700**	transcription factor activity	75	488	0.042
Gill	Up				
	**GO:0030528**	transcription regulator activity	1	524	0.041
	**GO:0008152**	metabolic process	108	5,566	0.001
	**GO:0007154**	cell communication	10	1,772	0.019
	Down				
	**GO:0050896**	response to stimulus	26	678	0.011
	**GO:0042221**	response to chemical stimulus	12	217	0.003

### Identification of differentially expressed genes

**Figure 2:**
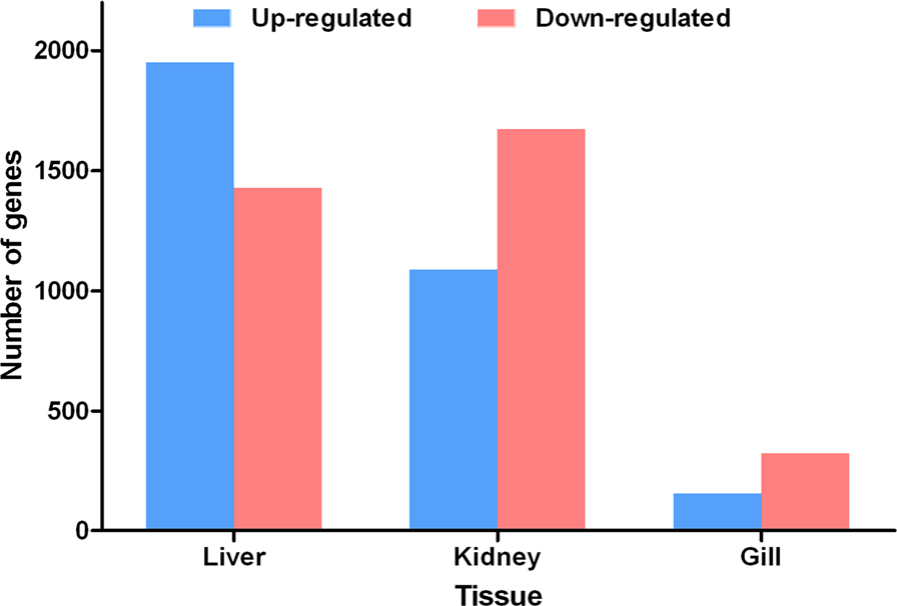
**DEGs in three tissues between AW and FW for *L. waleckii*.**

**Figure 3:**
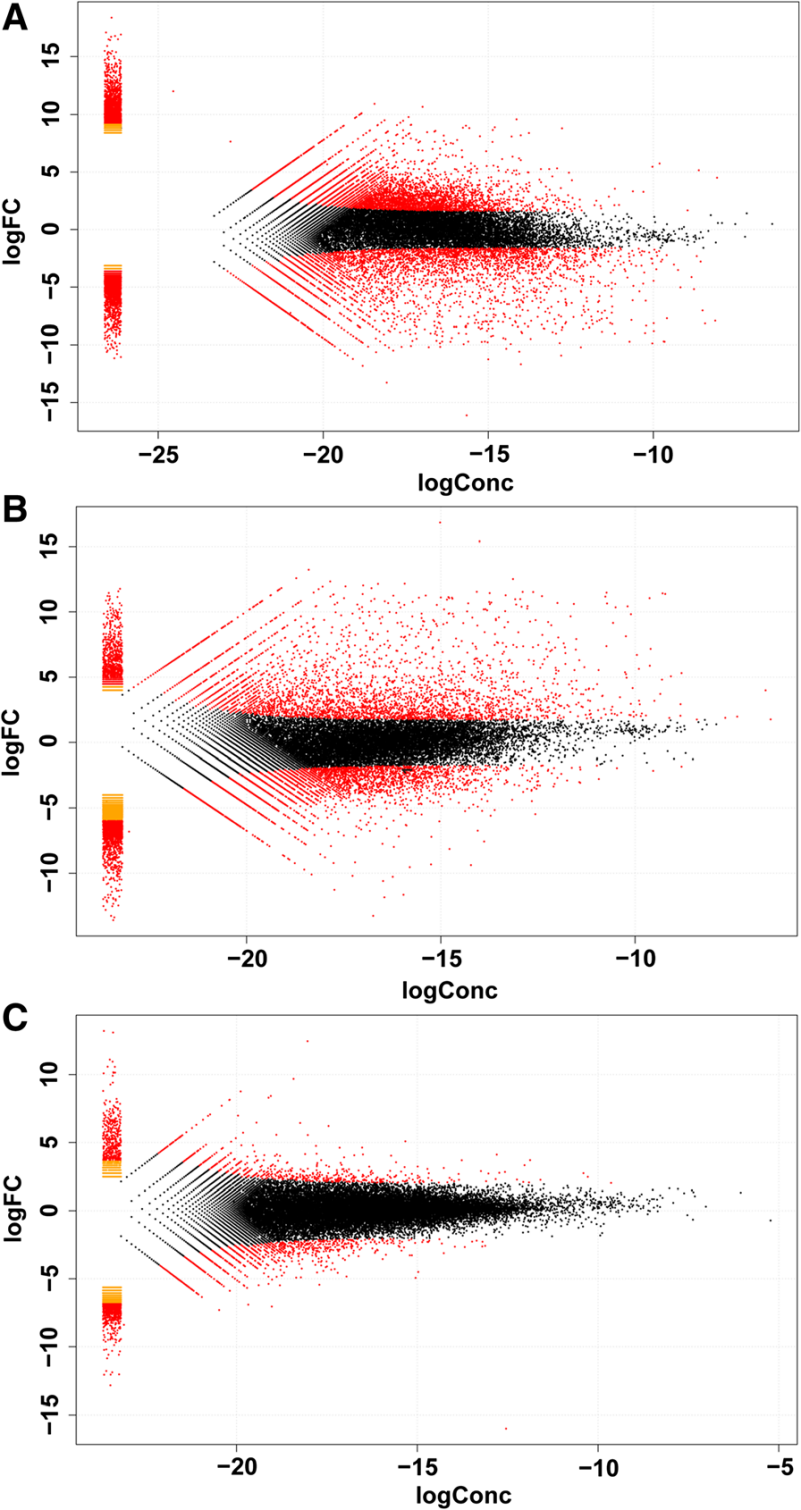
**M-A plots showing gene expression in three tissues. (A)** M-A plot showing gene expression in liver; **(B)** M-A plot showing gene expression in kidney; **(C)** M-A plot showing gene expression in gill.

**Figure 4:**
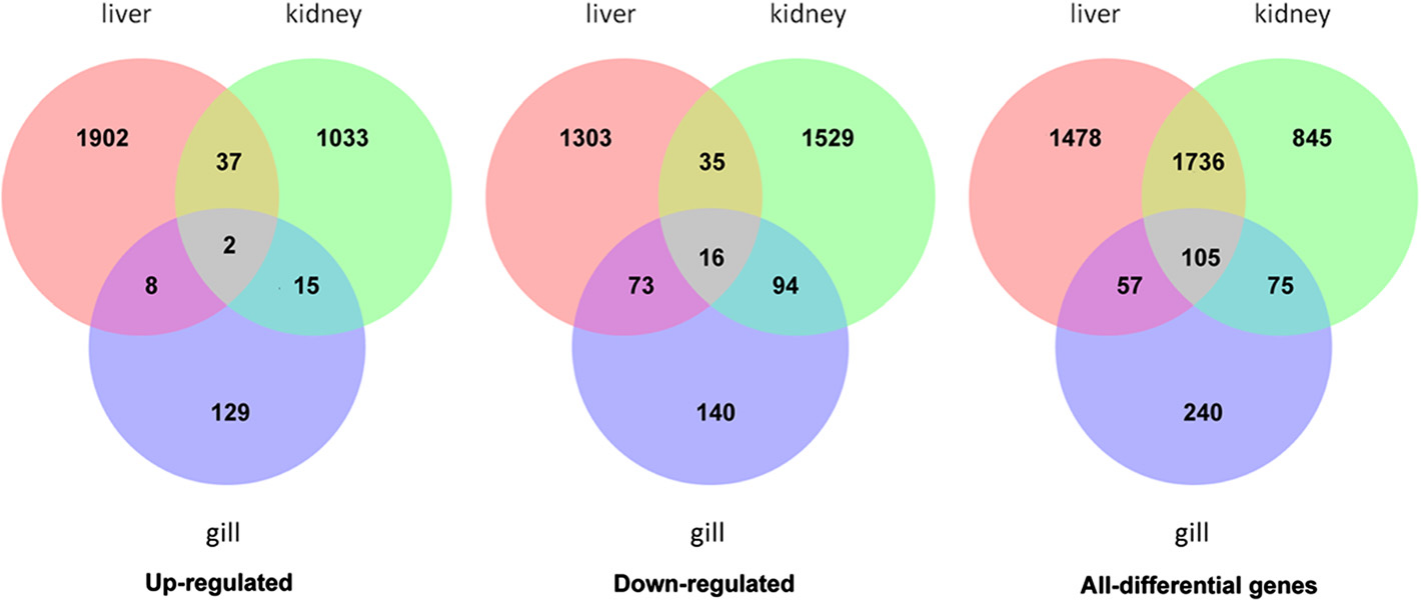
Venn diagram of DEGs among three tissues.

We found 477, 2,761 and 3,376 DEGs in the gill, kidney, and liver, respectively, of AW population compared to FW population with FDR ≤ 0.01 and fold-change ≥ 2 (Figure 2). M-A plots were drafted using “eps” format files as shown in Figure 3. Of these differentially expressed genes, 154, 1,087, 1,949 genes showed higher expression in gill, kidney, and liver of the AW population, respectively; and 323, 1,674, 1,427 genes showed higher expression in gill, kidney, and liver of the FW population, respectively. Of these, 127, 64, 314 genes were exclusively expressed in gill, kidney, and liver of the AW population, and 85, 335, 125 genes were exclusively expressed in in gill, kidney, and liver of the FW population (Additional file 1: Table S1). Venn diagram of the DEGs illustrated that majority of these genes were not shared in three tissues, suggesting that the mechanism and pathways in response to alkaline stress are significant different in gill, kidney, and liver (Figure 4).

**Figure 5:**
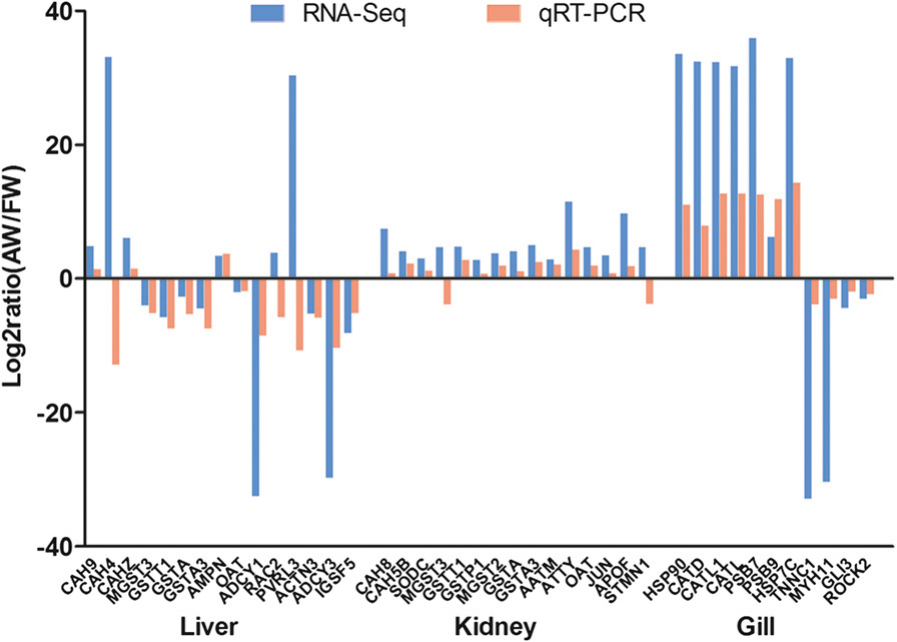
**Differentially expressed genes validated by qRT-PCR.** Comparison between RNA-Seq results and qRT-PCR validation results. X-axis shows genes in three tissues validated in this study; Y-axis shows Log_2_Ratio of expression of AW (alkaline water) versus FW (fresh water).

To validate RNA-Seq results, 35 genes with high level of significance or important stress-responding functions were selected for qRT-PCR analysis with beta-actin as reference gene. Primers for all genes are listed in Additional file 2: Table S2. Overall, the expression patterns of 30 genes were in agreement across the RNA-Seq and qRT-PCR analyses with minor differences in the expression level (Figure 5). There were only 5 genes that not showed the consistency of expression in the two assays. Thus, these genes showed similar patterns of mRNA abundance in RNA-Seq analysis and qRT-PCR, validated the genome-wide expressed profiling in gill, kidney, and liver in response to AW stress.

### Functional analysis on differential expressed genes in gill

In response to AW stress, we observed significant gene enrichment of several Gene Ontology (GO) terms in gill that related to stress response. These GO terms include “transcription regulator activity (GO:0030528)”, “metabolic process (GO:0008152)” and “cell communication (GO:0007154)” in up-regulated genes, and “response to stimulus (GO:0050896)” in the down-regulated genes. Notably, there are a total of 26 DEGs in the category of “response to stimulus”. Detailed analysis revealed that 12 genes are related to “response to stress (GO:0006950)”, 12 genes are related to “response to chemical stimulus (GO:0042221)”, 7 genes are related to “response to external stimulus (GO:0009605)” and 6 genes are related to “cellular response to stimulus (GO:0051716)”.

We further investigated those highly DEGs in gill, and observed that 7 heat shock protein genes, 4 Cathepsin genes and 3 proteasome subunit genes are highly up-regulated in gill of AW (Additional file 1: Table S1). Heat shock proteins target damaged proteins to the proteasome to prevent accumulation of dysfunctional proteins and to recycle peptides and amino acids [23]. This result suggested that the high level of autophagy occurred in gill under AW stress.

In the IPA analysis, we observed significant Kyoto Encyclopedia of Genes and Genomes (KEGG) pathway enrichment of the differential expressed genes in gill in response to AW environment stress. “Eukaryotic Initiation Factor 2 (EIF2) Signaling”, “Regulation of eIF4 (Eukaryotic Initiation Factor 4) and p70S6K (P70S6 kinase) Signaling” and “mTOR (Mammalian Target of Rapamycin) Signaling” were listed in the top enriched pathways, and all of them had been reported that played essential roles on stress response and tolerance. Eukaryotic Initiation Factor 2 (EIF2) is a GTP (Guanosine Triphosphate)-binding protein that escorts the initiation-specific form of Met-tRNA (Met-tRNAi) onto the ribosome, it also plays a role in identifying the translational start site. EIF2 signaling is the protein synthesis pathway in eukaryotic organisms. Because protein synthesis is energetically costly, stressed cells usually inhibit this process to devote resources to stress responses. In many cases EIF2α phosphorylation is a biological response that facilitates cells to cope with stressful environments by down-regulation of protein synthesis [24,25]. mTOR is a serine/threonine kinase distributed within two protein complexes (mTORC1 and mTORC2) in the cell [26], which plays important roles in response to stress, including activation of the autophagy [27] and modulation of protein synthesis [28]. These responses can conserve energy and promote survival during prolonged periods of stress [19]. “Regulation of eIF4 and p70S6K Signaling” pathway has similar functions on protein synthesis regulation. p70s6k has been considered an mTOR activation mirror and a marker of increased protein synthesis induced by stress and stimulation [29].

According to above evidences from GO and KEGG pathway analysis, we hypothesized that high alkaline stress suppressed protein synthesis and increased the level of autophagy in gill of *L. waleckii* in Dali Nor, which could conserve energy and provide sufficient amino acids and macromolecules for surviving in high alkaline environment.

### Functional analysis on differential expressed genes in liver

In response to AW stress, we observed significant gene enrichment under several GO terms in liver, including “oxidoreductase activity (GO:0016491) (52 genes)” and “transporter activity (GO:0005215) (139 genes)” in up-regulated genes, and “transferase activity (GO:0016740) (201 genes)”, “electron carrier activity (GO:0009055)” and “oxidoreductase activity (GO:0016491) (132 genes)” in the down-regulated genes. The genes in the molecular function category of “oxidoreductase activity” and “electron carrier activity” are widely studied and recognized to associate with oxidative stress and adaptation on environmental stimuli in coupling with mitochondrial functions. For instance, Mitchell *et al.* performed genome-wide gene expression profiling on two model microorganisms, *Escherichia coli* and *Saccharomyces cerevisiae*, in response to environmental stimuli, showed significant functional enrichment of oxidative stress categories, including oxidoreductase activity [30]. The expression profiling studies on teleost species also showed similar response that a cluster of genes of oxidoreductase activity differential expressed in response to environmental stress, such as temperature stress and confinement stress [31,32]. Here we identified 6 oxidoreductase genes differentially expressed in AW and FW environment. Genes in the category of “transporter activity” are in charge of the movement of substances, such as macromolecules, small molecules and ions, etc. They were significantly enriched in the sub-terms of “substrate-specific transmembrane transporter activity” and “ion transmembrane transporter activity” in this study, suggesting their important roles in regulating homeostasis of various substrates in response to environmental stress, which were consistent with those previous reports on stress adaptation and resistance of many organisms. For instance, amino acid and ion transmembrane transporters were reported to be essential factors to salt and osmotic stress response in many plants [33,34], as well as in many aquatic animals including mollusks [35,36] and teleosts [37,38] etc. We further inspected those highly DEGs in liver and confirmed that 128 accessions encoding various transporter proteins or solute carrier (SLC) family members, suggesting they were important regulators in response to alkaline stress in liver of *L. waleckii*. IPA pathway enrichment analysis on those DEGs in liver showed significant pathway enrichment on “Superpathway of cholesterol biosynthesis”, suggesting the cholesterol synthesis had been significantly induced under the severe environmental stress in the liver of *L. waleckii*.

### Functional analysis on differential expressed genes in kidney

Kidney is the essential organ which serves homeostatic functions such as the regulation of electrolytes, maintenance of acid–base balance, and salt and water balance in the body. From those 2,761 DEGs in kidney, we identified significant enrichment on several GO terms, including “oxidoreductase activity (GO:0016491) (150 genes)”, “transferase activity (GO:0016740) (165 genes)”, “transporter activity (GO:0005215) (77 genes)”, “electron carrier activity (GO:0009055) (32 genes)”, “enzyme regulator activity (GO:0030234) (56 genes)”, “response to stimulus (GO:0050896) (91 genes)” in the up-regulated genes, and “oxidoreductase activity (GO:0016491) (41 genes)”, “transporter activity (GO:0005215) (124 genes)”, “enzyme regulator activity (GO:0030234) (93 genes)”, “transcription factor activity (GO:0003700) (75 genes)” in the down-regulated genes. The enrichment profile is similar to those DEGs in liver on “oxidoreductase activity”, suggesting that both tissues were facing oxidative stress caused by environmental stimuli, and the genes with oxidoreductase activity changed their expression to adapt the changes. The genes of “transporter activity” were enriched in both up- and down-regulated genes. A significant portion of these transporters are substrate-specific transmembrane transporters (GO:0022891). The active transmembrane transporters are much more than those passive transmembrane transporters, which indicate that active transporters play the essential roles to transport specific substrates such as ion and organic acid across membranes under severe osmotic and alkaline stress with great energy consumption than those in FW environment. The increased energy requirement leads to active proteolysis in the kidney, which can be demonstrated by observed up-regulation of genes encoding various aminotransferases, including tyrosine aminotransferase, aspartate aminotransferase, ornithine aminotransferase, and alanine aminotransferase, etc. Other than aminotransferases, abundant genes under the term of “transferase activity” are significantly enriched in up-regulated genes, which are mainly comprised of “transferring one-carbon groups”, “transferring acyl groups”, “transferring glycosyl groups”, “transferring phosphorus-containing groups”, suggesting their important roles in response to environmental stress in AW. One of well-studied transferase families is glutathione S-transferase (GST) gene family, which have been confirmed their essential functions in protection against oxidative stress caused by various stress from toxic heavy metal ions [39-41], osmotic imbalance [42], salinity [43] and pH change [44]. GSTs have been even used as biomarkers for environmental pollution and toxins monitoring recently [45,46]. In the biological process, we identified 47 DEGs that belong to the subcategory of “response to chemical stimulus” in those 91 up-regulated genes of “response to stimulus” in kidney, corresponding to the essential roles of kidney in response to external and endogenous chemical stresses in AW environment.

### Expression of the genes under positive selection

Previous dN/dS analysis on transcriptome of *L. waleckii* from Dali Nor Lake revealed that there were 61 genes experienced strong positive selection under severe environmental stress [7]. We investigated the genes under strong positive selection and found that significant portions of these genes were also expressed differentially under AW and FW environment (Table 4). For instance, we identified 5 carbonic anhydrase genes, 2 superoxide dismutase genes, 5 glutathione S-transferase genes, 3 aminopeptidase N genes, and 2 perforin-1 genes from the DEG list of liver, and identified 4 carbonic anhydrase genes, 2 superoxide dismutase genes, 8 glutathione S-transferase genes, 3 aminopeptidase N genes, and 2 Perforin-1 genes from the DEG list of kidney. Obviously, a number of genes that retain specific nucleotide changes under strong positive selection also change their expression profiles under severe alkaline stress, as well as osmotic, salt and heavy metal stress in the Dali Nor lake.

**Table 4:** Differentially expression of positively selected genes

**Genes**	**Gene ID**	**Differentially expression tissues**		
Integrin alpha-X	sp|P20702|ITAX_HUMAN		kidney	liver
Polymeric immunoglobulin receptor	sp|P15083|PIGR_RAT		kidney	liver
T-cell receptor beta chain T17T-22	sp|P11364|TCB_FLV		kidney	liver
Cytokine receptor common subunit gamma	sp|Q95118|IL2RG_BOVIN		kidney	liver
Transposable element Tcb1 transposase	sp|P35072|TCB1_CAEBR		kidney	liver
B-cell antigen receptor complex-associated protein alpha chain	sp|P11911|CD79A_MOUSE		kidney	liver
Carbonic anhydrase 4	sp|P48284|CAH4_RAT		kidney	liver
Interleukin-8	sp|P08317|IL8_CHICK			liver
Extracellular superoxide dismutase [Cu-Zn]	sp|O09164|SODE_MOUSE		kidney	
Stonustoxin subunit beta	sp|Q91453|STXB_SYNHO	gill		liver
Ig mu chain C region membrane-bound form	sp|P01873|MUCM_MOUSE		kidney	liver
Transposable element Tc1 transposase	sp|P03934|TC1A		kidney	liver
Perforin-1	sp|P14222|PERF_HUMAN		kidney	liver
Midnolin	sp|Q5EB28|MIDN_XENTR		kidney	liver
Apolipoprotein B-100	sp|P04114|APOB_HUMAN	gill	kidney	liver
Disabled homolog 2-interacting protein	sp|Q3UHC7|DAB2P_MOUSE		kidney	liver
Complement factor H	sp|P06909|CFAH_MOUSE		kidney	liver
Glutathione S-transferase A	sp|P30568|GSTA_PLEPL		kidney	liver
Complement factor B	sp|P81187|CFAB_BOVIN		kidney	liver
Caspase-1	sp|P55867|CAS1B_XENLA			liver
Myb-binding protein 1A-like protein	sp|Q6DRL5|MBB1A_DANRE			liver
Plexin-C1	sp|O60486|PLXC1_HUMAN		kidney	
Interferon-induced very large GTPase 1	sp|Q7Z2Y8|GVIN1_HUMAN			liver
RNA-directed DNA polymerase from mobile element jockey	sp|P21329|RTJK_DROFU	gill	kidney	liver
Ig heavy chain V region 5A	sp|P19181|HV05_CARAU		kidney	liver
Aminopeptidase N	sp|P15684|AMPN_RAT		kidney	liver
UPF0577 protein KIAA1324-like	sp|A8MWY0|K132L_HUMAN		kidney	
UHRF1-binding protein 1-like	sp|Q6NRZ1|UH1BL_XENLA			liver
Nuclear factor 7, ovary	sp|Q91431|NF7O_XENLA		kidney	
Ceramide synthase 2	sp|Q3ZBF8|CERS2_BOVIN		kidney	liver
Laminin subunit alpha-3	sp|Q16787|LAMA3_HUMAN		kidney	

## Conclusion

We performed comparative transcriptome profiling study on *L. waleckii* inhibiting in alkaline water of Dali Nor lake and in fresh water of Ganggeng Nor lake, and identified a relatively large number of genes that displayed distinct differences on their expression in gill, liver and kidney. Further analysis revealed that several well-known functional categories of genes and signaling pathway, which are associated with stress response and extreme environment adaptation, had been significantly enriched, including the functional categories of “response to stimulus”, “transferase activity”, “transporter activity” and “oxidoreductase activity”, etc., and signaling pathways of “mTOR signaling”, “EIF2 signaling”, “superpathway of cholesterol biosynthesis”, etc. We also identified significantly DEGs in three tissues, encoding important modulators on stress adaptation and tolerance, including carbonic anhydrases, heat shock proteins, superoxide dismutase, glutathione S-transferases, aminopeptidase N, and aminotransferases. Overall, this study demonstrated that transcriptome changes in *L. waleckii* played a role in adaptation to complicated environmental stress in the highly alkalized Dali Nor lake. The results set a foundation for further analyses on alkaline-responsive candidate genes, which would help us understand teleost adaptation under extreme environmental stress and ultimately benefit future breeding for alkaline-tolerant fish strains.

## Methods

### Ethics statement

This study was approved by the Animal Care and Use committee of Centre for Applied Aquatic Genomics at Chinese Academy of Fishery Sciences.

### Fish sampling

Ten individuals (five males, five females) of *L. waleckii* inhabiting alkaline water were sampled at north shore of Dali Nor lake, Inner Mongolia, China (43^o^22′43"N, 116^o^39'24"E). Nine individuals (five males, four females) inhabiting fresh water were sampled at west shore of Ganggeng Nor lake (43^o^17'48"N, 116^o^53'27"E). Both groups weighted ranging from 130 grams to 150 grams. Liver, kidney and gill were dissected and collected, due to previous report that these organs play important role in hyperosmotic and hypersaline conditions [47]. Tissue samples were stored in RNAlater (Qiagen, Hilden, Germany) and transported to laboratory in Beijing at room temperature, then stored at −20°C prior to RNA extraction.

### RNA extraction and quality control

Total RNA was extracted from each tissue using TRIZOL Kit (Invitrogen, Carlsbad, CA, USA) with manufacturer’s instructions. RNA samples were then digested by DNase I to eliminate potential genomic DNA. Integrity and size distribution were checked on Bioanalyzer 2100 with RNA 6000 Nano Labchips (Agilent technologies, Santa Clara, CA, USA). Equal amounts of the high quality RNA samples from each tissue were then pooled for RNA-Seq.

### cDNA library construction and sequencing

RNA-Seq library preparation and sequencing was carried out by HudsonAlpha Genomic Services Lab (Huntsville, AL, USA) as previously described [48]. cDNA libraries were prepared with ∼2.5 μg of starting total RNA following the protocols of the Illumina TruSeq RNA Sample Preparation Kit (Illumina). The final library had an average fragment size of 270 bp and final yields of 400 ng. After KAPA quantitation and dilution, the library was sequenced on an Illumina HiSeq 2000 with 101 bp paired-end reads.

### Sequence data processing and *de novo* assembly

Adaptor sequences were trimmed and low quality reads were removed. Then read length less than 10 were removed. TRINITY was used to assemble all cleaned reads with default parameters [22] and generate reference sequences for comparative transcriptome study.

### Functional annotation of assembled contigs

The assembled transcriptome contigs were subjected to similarity search against NCBI non-redundant (nr) protein database using BLASTx with e-value cutoff of 1E-10. Gene name and description was assigned to each contig based on the top BLASTx hit with the highest score. Gene ontology (GO) analysis was conducted on assembled transcriptome using InterProScan (http://www.ebi.ac.uk/Tools/pfa/iprscan/) and integrated protein databases with default parameters. The GO terms associated with transcriptome contigs were then obtained for describing their biological processes, molecular functions and cellular components.

### Read mapping and differential gene expression analysis

All the cleaned reads were mapped to the assembled reference transcriptome by Bowtie [21], and about 84.1% of the reads can be mapped to the reference for each sample (Table 1). RSEM was then used to estimate and quantify the gene and isoform abundances according to the trinity assembled transcriptome. Finally, we used edgeR to normalize the expression levels in each of these samples and obtain the differentially expressed transcripts by pairwise comparisons [49].

### Quantitative reverse transcription-PCR (qRT-PCR)

qRT-PCR was used to validate the RNA-Seq results on randomly selected 30 gene accessions. The beta-actin gene was used as an internal reference, and primers were designed as below, forward primer: 5′- TGCAAAGCCGGATTCGCTGG -3′; reverse primer: 5′- AGTTGGTGACAATACCGTGC -3′. Briefly, qRT-PCR was performed in the optical 96-well plates with an ABI PRISM 7500 Real-time Detection System (Life Technology). The amplification was performed in a total volume of 15 μl, containing 7.5 μl 2X SYBR Green Master Mix reagent (Life Technology), 1 μl of cDNA (100 ng/μl), and 0.3 μl of 10 μM of each gene-specific primer. The PCR cycle was 50°C for 2 min, 95°C for 10 min, 40 cycles of 95°C for 15 s and 60°C for 1 min. All reactions were set up in triplicate including the negative controls with no template. To assess PCR efficiency, five 10-fold serial dilutions of a randomly selected cDNA sample were used on both the target genes and the reference gene to assess the PCR efficiency. After the PCR, data were analyzed with ABI 7500 SDS software. The comparative CT method (2^-∆∆CT^ method) was used to analyze the expression of the target genes. All data were given at levels relative to the expression of the beta-actin gene.

### IPA analysis

The genes differentially expressed in 3 tissues were further analyzed using the Ingenuity Pathway Analysis program (IPA; https://analysis.ingenuity.com) in order to identify the biochemical pathways affected. The IPA software contains the biological function, interaction, and information of a curated gene set and many biochemical pathways, identifying global canonical pathways, dynamical biological networks and functions from a given list of genes. Basically, the accession numbers of DEGs were uploaded into the IPA and compared with the genes included in each canonical pathway using the whole gene set as the background. All the pathways with one or more genes overlapping the candidate genes were extracted. During IPA analyses, each of the pathways was assigned a P value from Fisher’s exact test, denoting the probability of overlap between the pathway and the input genes.

### Availability of supporting data

All supporting files have been deposited to LabArchives entitled ‘amur ide DGE’ with a DOI of 10.6070/H4PZ56RJ (https://mynotebook.labarchives.com/share/xujian/Mi42fDIxOTY2LzIvVHJlZU5vZGUvMzc5NDY5ODU2MHw2LjY=).

## Competing interests

The authors declare that they have no competing interests.

## Authors’ contributions

JX and QL contributed equally and their contribution accounts for the major part of this study. PX conceived the study, and drafted the manuscript. JX and QL worked on sample collection and RNA sequencing, and participated in major bioinformatic analysis and manuscript preparation. LX and JL worked on DEG pipeline development. SW and YJ participated in gene pathway analysis. ZZ, YZ and CD participated in sample collection. XS participated in discussions and provided advices. All authors read and approved the final manuscript.

## Supplementary Material

Additional file 1: Table S1 DGE in three different tissues. Click here for file

Additional file 2: Table S2 Primers used in validation of DGEs. Click here for file
